# BMP10 reflects pre-capillary pulmonary hemodynamics: association of biomarkers and hemodynamic parameters in pulmonary hypertension

**DOI:** 10.1007/s00392-024-02546-8

**Published:** 2024-09-19

**Authors:** Elisa Hennings, Stefanie Aeschbacher, Michael Coslovsky, Rebecca E. Paladini, Gian Voellmin, Maurin Lampart, André Ziegler, Christian Müller, David Conen, Christine S. Zuern, Michael Kühne, Stefan Osswald, Otmar Pfister

**Affiliations:** 1 Cardiovascular Research Institute Basel, University Hospital Basel, University of Basel, Basel, Switzerland; 2 Cardiology, University Hospital Basel, University of Basel, Petersgraben 4, 4031 Basel, Switzerland; 3 Department of Clinical Research, University Hospital Basel, University of Basel, Basel, Switzerland; 4 Roche Diagnostics International AG, Rotkreuz, Switzerland; 5 Population Health Research Institute, McMaster University, Hamilton, Canada

**Keywords:** Biomarker, BMP10, NT-proBNP, Pulmonary hypertension, Right heart catheterization

## Abstract

**Background and aims:**

The role of biomarkers in diagnosing pulmonary hypertension (PH) and distinguishing between pre- and post-capillary PH remains poorly understood. We aimed to identify biomarkers with a strong association with mean pulmonary arterial pressure, mPAP (PH diagnosis) and pulmonary vascular resistance, PVR (pre-capillary component), but not with pulmonary arterial wedge pressure, PAWP (post-capillary component).

**Methods:**

Blood samples were collected in patients undergoing right heart catheterization within a prospective cross-sectional study. Biomarkers measured included BMP10, NT-proBNP, ANG2, ESM1/endocan, FGF23, GDF15, IGFBP7, IL6, MyBPC3, proC3, and proC6/endotrophin. Primary outcomes were mPAP, PVR, and PAWP, while secondary outcomes included PH diagnosis (mPAP > 20 mmHg) and elevated PVR (> 2 Wood units). Multivariable linear and logistic regression models were used to assess the relationship between biomarkers and outcomes.

**Results:**

Of the 127 patients included (age 66 ± 13 years, 54% female), 73% were diagnosed with PH. BMP10, NT-proBNP, ANG2, MyBPC3, and FGF23 showed a strong association with mPAP (*p* < 0.001). BMP10 and NT-proBNP were strongly associated with PVR (*p* < 0.001), while NT-proBNP and ANG2 were strongly associated with PAWP (*p* < 0.001). NT-proBNP had the strongest association with the diagnosis of PH (area under the curve = 0.76). BMP10 was the only biomarker associated with elevated PVR (OR 1.60, 95%CI 1.01–2.54, *p* = 0.04) but not with PAWP (*p* = 0.86).

**Conclusions:**

Several biomarkers were strongly associated with mPAP, PAWP, and PVR. BMP10 was the only biomarker strongly associated with mPAP and PVR, but not with PAWP, thus reflecting the pre-capillary PH component. Measurement of BMP10 along with NT-proBNP may aid in diagnosing PH.

**Graphical abstract:**

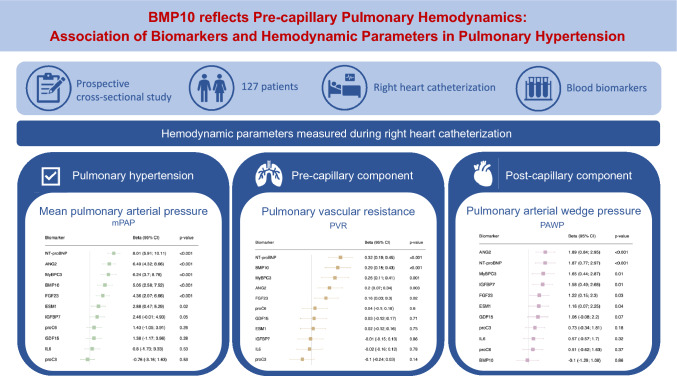

**Supplementary Information:**

The online version contains supplementary material available at 10.1007/s00392-024-02546-8.

## Introduction

Pulmonary hypertension (PH) is a severe and complex disease characterized by abnormally high pressure in the pulmonary arteries that eventually leads to right ventricular dysfunction and failure [1]. In PH, both an elevation of left atrial filling pressure (post-capillary component) and an increase in pulmonary vascular resistance (pre-capillary component) contribute to the elevation of the mean pulmonary arterial pressure (mPAP). Echocardiography may detect characteristic signs of PH such as right ventricular pressure overload and dysfunction, but lacks sufficient sensitivity to diagnose PH [1]. Another important shortcoming of echocardiography is its inability to identify and distinguish specific hemodynamic PH profiles, in particular the pre-capillary component of PH. Therefore, invasive hemodynamic assessment by right heart catheterization (RHC) is mandatory to confirm the diagnosis of PH and to unravel the respective contributions of pre- and post-capillary hemodynamic components [1].

Blood-based biomarkers might help to better phenotype PH and have the potential to function as a non-invasive tool in addition to echocardiography in the diagnostic algorithm to define who should receive a RHC.To date, N-terminal prohormone of B-type natriuretic peptide (NT-proBNP) is the only biomarker recommended by international guidelines in the setting of PH [1]. In PH, NT-proBNP serum levels correlate with right ventricular function and hemodynamic parameters [2]. However, for hemodynamic phenotyping NT-proBNP is of limited use because it reflects both left atrial filling pressure, which can be assessed by the pulmonary arterial wedge pressure (PAWP), and pulmonary vascular resistance (PVR) [2]. NT-proBNP therefore lacks discriminative ability between the post-capillary (PAWP) and the pre-capillary component (PVR) in PH. For this purpose, a discriminative biomarker specifically reflecting PVR, but not left atrial filling pressure (PAWP) would be highly useful.

The aim of this study was to identify biomarkers with a strong association with mPAP and PVR but little to no association with left atrial filling pressure (measured by PAWP). Therefore, we prospectively assessed the association of a preselected set of candidate biomarkers with hemodynamic parameters (i.e., mPAP, PVR, PAWP) in consecutive patients undergoing RHC.

## Methods

### Study design, setting, and participants

We analyzed patients with suspected or established PH (also those on treatment) undergoing RHC within a prospective, single-center, cross-sectional study conducted between May 2021 and November 2022 at the University Hospital Basel, Switzerland. Patients aged ≥ 18 years were eligible and were consecutively screened. We excluded patients who had experienced atrial fibrillation in the previous 6 months because the tested biomarkers can be influenced by rhythm (i.e., bone morphogenetic protein 10 [BMP10], NT-proBNP) [3, 4]. The study protocol was approved by the Ethics Committee Northwest and Central Switzerland (EKNZ, project-ID 2021–00125), and the study complied with the Declaration of Helsinki. We obtained written informed consent from each patient.

### Blood sampling and other study variables

We obtained venous blood samples from study participants at the beginning of the RHC. Blood samples were centrifuged, aliquoted into cryotubes, and stored at −80 °C in a biobank. Biomarker concentrations were measured centrally (Roche Diagnostics, Penzberg, Germany) using a cobas e601 analyzer. The following biomarkers were assessed: Angiopoietin 2 (ANG2), BMP10, endothelial cell specific molecule 1 (ESM1, endocan), fibroblast growth factor 23 (FGF23), growth differentiation factor 15 (GDF15), insulin-like growth factor-binding protein 7 (IGFBP7), interleukin 6 (IL6), myosin binding protein C3 (MyBPC3), NT-proBNP, N-terminal type III collagen propeptide (proC3), and fragment of C-terminal type VI (α3) collagen (proC6, endotrophin). We selected a comprehensive set of biomarkers to capture the diverse pathophysiological processes involved in PH, including cardiac function and stress, vascular remodeling and endothelial function, metabolic regulation, inflammation and growth factors, extracellular matrix and fibrosis. For the analysis of GDF15, IL6, and NT-proBNP, the commercial IVDR (in vitro diagnostic medical device regulation) products were used. For the newer biomarkers (ANG2, BMP10, ESM1, FGF23, IGFBP7, MyBPC2, proC1, and proC6), non-commercial robust prototype assays (RPA) were used that are also based on the Elecsys® Electro-ChemiLuminescence (ECL) technology. The assays employ a quantitative sandwich principle, where the first-monoclonal antibody specifically binds circulating protein biomarkers as a capture antibody and a ruthenylated second-monoclonal antibody binds the biomarker as a detection antibody. Recombinant biomarkers are used to normalize the measurements across the runs. The laboratory personnel were unaware of any clinical information.

We used standardized questionnaires to collect data from patient records on the patients’ characteristics, lifestyle, medical history, medication, and echocardiographic data if available. In addition, we calculated the body mass index (BMI; kg/m^2^).

### Right heart catheterization and outcome measures

RHC was performed by experienced cardiologists using a standardized protocol. The mid-thoracic level in supine position of the patient was the zero reference level. Vascular access was gained by the jugular or femoral vein. Pressures in the right atrium, pulmonary artery, and pulmonary artery wedge position were measured by a wedge pressure balloon floating catheter (Braun®). All pressure measurements were performed at the end expiration. Saturation was taken in the wedge position to confirm an accurate PAWP. Systemic and mixed venous O2 content were determined by blood sampling and cardiac output was calculated using the Fick method.

Two physicians independently adjudicated the PH diagnosis using the hemodynamic variables measured during RHC. Physicians were not aware of the biomarker concentrations.

The primary outcome variables were mPAP, PVR, and PAWP. The secondary outcome variables were the diagnosis of PH (“yes” if mPAP > 20 mmHg, “no” if mPAP ≤ 20 mmHg) and elevated PVR (“yes” if PVR > 2 Wood units [WU] equals 160 dyn*s*cm^−5^, “no” if PVR ≤ 2 WU). PH diagnosis and elevated PVR were defined according to the European Society of Cardiology (ESC) PH guidelines of 2022 [1].

### Statistical analyses

We present the baseline characteristics overall and stratified by PH (yes/no). Categorical variables, indicated as numbers (percentages), were compared using Chi-square tests. Continuous variables, indicated as mean ± standard deviation (SD) or median [interquartile range], were compared using t test or Wilcoxon rank sum test as appropriate.

We built linear-regression models to determine the association of biomarker concentration with hemodynamic parameters (mPAP, PVR, PAWP). One model was unadjusted. A second model was adjusted for patient age and sex. A third model was additionally adjusted for BMI. We present the results as β coefficient with corresponding 95% confidence intervals (CI). Assumptions including normal error distribution and homoscedasticity were checked by examining model residuals. We calculated the R^2^ and Akaike information criterion (AIC) for each model. A higher R^2^ indicates a higher proportion of variance in the outcome that is explained by the predictors. Lower AIC values indicate better model fit for a given set of data. For graphical presentation, we constructed scatterplots and used the locally estimated scatterplot smoothing (LOESS) method to fit a smooth curve through the points of the scatterplot. We added the linear-regression line of the model adjusted for age, sex, and BMI for an “average” patient (continuous variables were set to the mean and categorical variables to the most frequent category). The Spearman correlation coefficients were calculated and added to the plots.

We fitted logistic regression models to determine the association of biomarker concentration and PH (yes/no) and elevated PVR (yes/no). Models were adjusted for age, sex, and BMI. Results are presented as odds ratio (OR) and 95% CI. We computed the Brier score, AIC, and Nagelkerke’s R^2^. The Brier score is a measure of calibration, that is, how well do model predictions fit the observed data. It ranges from 0 to 1, with lower values indicating greater accuracy of the predictions and better calibration. A higher Nagelkerke’s R^2^ (i.e., closer to 1) indicates a better goodness of fit. For graphical presentation, we constructed receiver operating curves (ROC) and calculated the area under the curve (AUC). For logistic regression the AUC-ROC is equivalent to the concordance statistic, a measure of discrimination. A higher AUC indicates superior discrimination ability, implying that the model is superior at distinguishing between positive and negative cases. We plotted the predicted probabilities for PH according to the different biomarkers. We included and tested interaction terms for sex and age with the relevant biomarkers to check whether sex or age had an effect on the diagnosis of PH in the different biomarker models. A further exploratory analysis involved variable selection by backwards-forwards selection as well as based on the lowest AIC of all possible biomarker model combinations (R package “glmulti”) to identify the best multibiomarker model for the diagnosis of PH. For the best model, we calculated the AIC, AUC, Brier score, and Nagelkerke’s R^2^. We then performed a likelihood ratio test to compare the multibiomarker model to the best univariable biomarker model.

All biomarkers were log-transformed because their distributions were skewed. They were then standardized in z-scores for the purpose of comparison, thus beta coefficients reflect the change in standard deviation of log-transformed biomarker concentration, if not indicated otherwise. Correlations between biomarkers were assessed by the Spearman correlation coefficient, and multicollinearity was assessed by the variance inflation factor (VIF). For our analyses, we used all cases with complete datasets. Because we anticipated very few missing values, we excluded patients with missing biomarker concentrations or covariates from the analysis. Due to the exploratory nature of the analysis, we performed no correction for multiple testing and interpreted p values as a continuous variable adding to the evidence against the relevant null hypothesis. The presented p values are two-sided. All analyses were performed using the statistical software R version 4.2.1 (2022-06-23, R Core Team).

## Results

### Study population

A total of 132 consecutive patients undergoing RHC because of suspected or established PH were included in the study. Three patients were excluded due to missing biomarker concentrations and two patients were excluded due to atrial fibrillation during the procedure, leaving 127 patients for this analysis. Table 1 shows the baseline characteristics overall and stratified by PH. Mean (± SD) age of the study population was 66 ± 13 years, and 54% of the patients were female. Compared to patients without PH, patients with PH had a slightly higher BMI and were more likely to have a history of chronic obstructive pulmonary disease (COPD) or lung emphysema. Median left ventricular ejection fraction (LVEF) measured by echocardiography was 60% [IQR 55%, 63%] with no strong difference between groups. Within the study cohort, mean (± SD) pulmonary arterial pressure (mPAP) was 30.8 ± 13.7 mmHg, median pulmonary vascular resistance (PVR) was 215.6 dyn*S*cm^−5^ [IQR 135.2, 349.3], and mean pulmonary arterial wedge pressure (PAWP) was 12.5 ± 6.4 mmHg. PH was diagnosed in 93 (73%) patients. Of all included patient, 34 (27%) patients had no PH, 54 (43%) patients had pre-capillary PH, 11 (9%) patients had post-capillary PH, 22 (17%) patients had combined pre- and post-capillary PH, and 6 (5%) patients had unclassified PH (Fig. S1). The clinical classification is shown in Fig. S2.

**Table 1:** Baseline characteristics overall and stratified by pulmonary hypertension (yes/no)

Characteristics	Overall	Pulmonary hypertension		
		No	Yes	*p* value
Number of patients	127 (100)	34 (27)	93 (73)	
Age, years	65.66 ± 12.71	62.47 ± 13.86	66.83 ± 12.13	0.09
Female sex, *n* (%)	68 (53.5)	19 (55.9)	49 (52.7)	0.91
Body mass index, kg/m^2^	27.64 ± 5.87	25.77 (4.22)	28.32 ± 6.25	0.03
Smoking status, *n* (%)				0.31
Never	54 (42.5)	16 (47.1)	38 (40.9)	
Past	51 (40.2)	15 (44.1)	36 (38.7)	
Current	22 (17.3)	3 (8.8)	19 (20.4)	
History of atrial fibrillation, n (%)	11 (8.7)	1 (2.9)	10 (10.8)	0.30
History of heart failure, n (%)	45 (35.4)	9 (26.5)	36 (38.7)	0.29
History of hypertension, n (%)	72 (56.7)	17 (50.0)	55 (59.1)	0.47
History of diabetes, n (%)	33 (26.0)	8 (23.5)	25 (26.9)	0.88
History of chronic obstructive pulmonary disease, n (%)	30 (23.6)	3 (8.8)	27 (29.0)	0.03
Lung emphysema, n (%)	31 (24.4)	2 (5.9)	29 (31.2)	0.01
History of pulmonary embolism, n (%)	30 (23.6)	6 (17.6)	24 (25.8)	0.47
Hypercholesterolemia, n (%)	38 (29.9)	10 (29.4)	28 (30.1)	1
Left ventricular ejection fraction (LVEF)	60 [55, 63]	60 [55, 64]	60 [55, 63]	0.92
Right heart catheterization:				
Mean pulmonary arterial pressure (mPAP), mmHg	30.81 ± 13.70	16.32 ± 2.67	36.11 ± 12.18	< 0.001
Pulmonary vascular resistance (PVR), dyn*S*cm^−5^	215.57 [135.20, 349.32]	117.81 [80.00, 148.45]	266.67 [187.00, 482.14]	< 0.001
Pulmonary arterial wedge pressure (PAWP), mmHg	12.45 ± 6.36	7.88 ± 2.98	14.12 ± 6.45	< 0.001
Cardiac index, L/min/m^2^	3.06 ± 0.92	3.30 ± 0.93	2.97 ± 0.90	0.08
Medication:				
Endothelin receptor antagonists	7 (5.5)	0 (0.0)	7 (7.5)	0.23
Phosphodiesterase 5 inhibitors	3 (2.4)	0 (0.0)	3 (3.2)	0.69
Guanylate-cyclase stimulators	3 (2.4)	0 (0.0)	3 (3.2)	0.69
Prostacyclin analogs	2 (1.6)	0 (0.0)	2 (2.2)	0.96

Values are given as mean ± standard deviation, median [interquartile range], or numbers (percentage). Missing value: LVEF (*n* = 1)

### BMP10 is the only biomarker associated with high pulmonary vascular resistance (PVR) independent of left atrial filling pressure

**Fig. 1:**
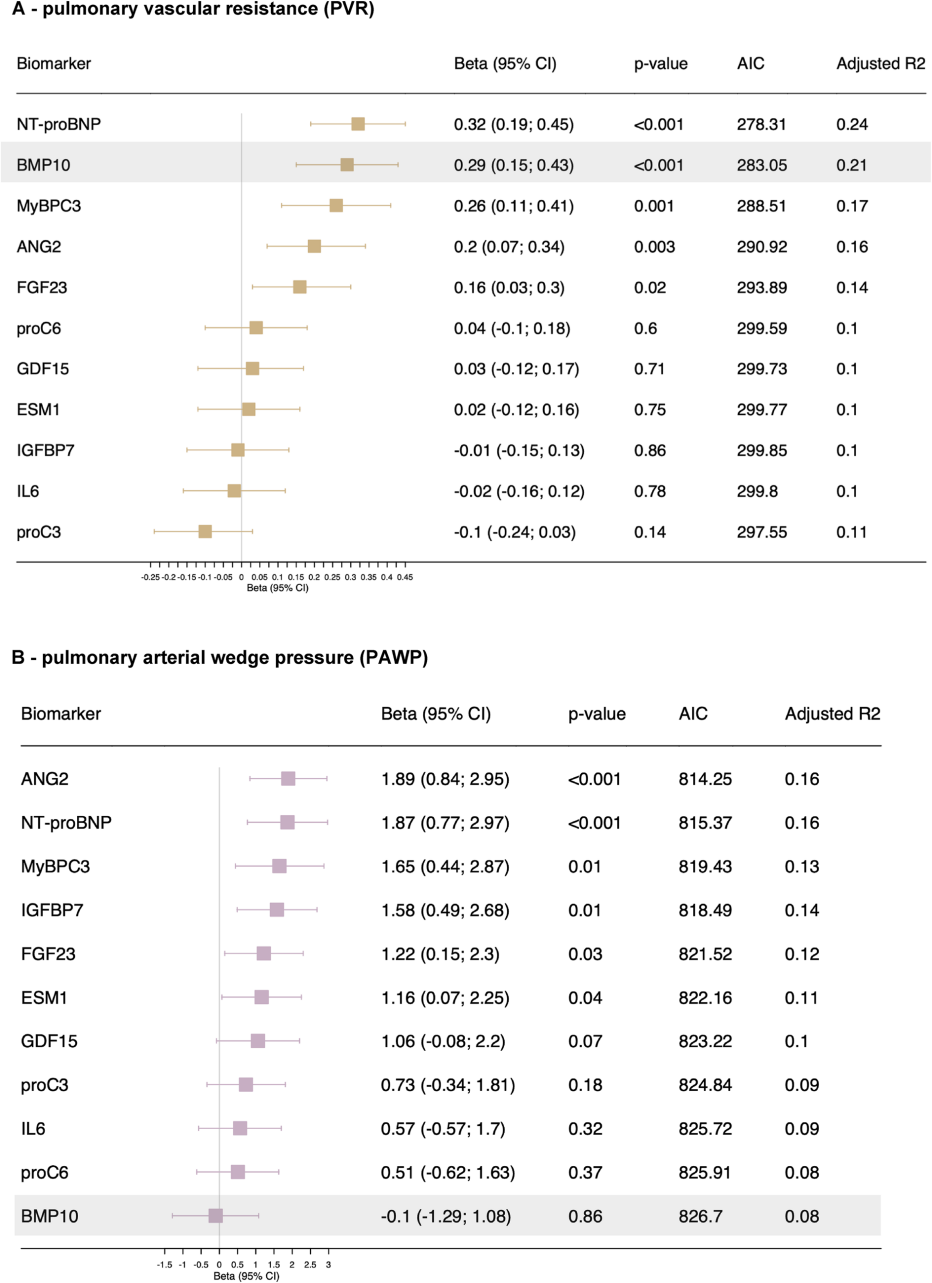
Association of biomarkers with pulmonary vascular resistance (PVR) and pulmonary arterial wedge pressure (PAWP). Linear regression of the respective biomarker model adjusted for age, sex, and body mass index for **A** PVR and **B** PAWP. All biomarkers are log-transformed and standardized. The outcome PVR was log-transformed as well. Models are sorted by β coefficients. *AIC* = Akaike information criterion

Because pulmonary arterial pressure depends on both PVR (pre-capillary component of mPAP) and left atrial filling pressure (post-capillary component of mPAP, measured by PAWP), we determined the association of biomarkers with these hemodynamic parameters in linear-regression models. For the parameter PVR, the biomarkers NT-proBNP, BMP10, MyBPC3, ANG2, and FGF23 demonstrated strong positive associations in the adjusted models for age, sex, and BMI, in descending order (Fig. 1A). Among these biomarkers, BMP10 was the only biomarker with a significant association of high PVR (> 2 WU equal to > 160 dyn*s*cm^-5^) with an OR of 1.60 (95% CI 1.01; 2.54), *p* = 0.04 (Table 2).

**Table 2:** Association of biomarker concentration and high pulmonary vascular resistance (PVR) yes/no, defined as > 2 WU equal to > 160 dyn*s*cm^-5^

Logistic regression	Age-, sex-, and BMI-adjusted model
	Standardized OR (95% CI), p value
ANG2	1.10 (0.74; 1.63), 0.64
BMP10	1.60 (1.01; 2.54), 0.04
ESM1	0.73 (0.49; 1.08), 0.11
FGF23	1.08 (0.73; 1.60), 0.71
GDF15	0.86 (0.58; 1.28), 0.46
IGFBP7	0.75 (0.51; 1.12), 0.16
IL6	0.80 (0.54; 1.19), 0.27
MyBPC3	1.17 (0.74; 1.84), 0.51
NT-proBNP	1.27 (0.84; 1.93), 0.25
proC3	0.95 (0.66; 1.38), 0.80
proC6	1.04 (0.70; 1.55), 0.83

*n* = 127 (PVR high no = 46, yes = 81 = event). All biomarkers are log-transformed and standardized

*BMI* body mass index, *CI* confidence interval, *OR* odds ratio, *WU* Wood units

For the hemodynamic parameter PAWP, which represents the left atrial filling pressure, ANG2, NT-proBNP, MyBPC3, IGFBP7, and FGF23 showed strong positive associations in the adjusted models for age, sex, and BMI, in descending order (Fig. 1B). Notably, no association with PAWP was found for BMP10 (standardized β = −0.10 [95% CI −1.29; 1.08], *p* = 0.86). Tables S1 and S2 show the calculations for the unadjusted and adjusted models (age and sex) for PVR and PAWP. Figures S3 and S4 show the scatterplots of biomarker values and PVR/PAWP, as well as the corresponding linear-regression lines and Spearman correlation coefficients.

### NT-proBNP exhibits the strongest association with mean pulmonary arterial pressure (mPAP) and best predicts pulmonary hypertension

**Fig. 2:**
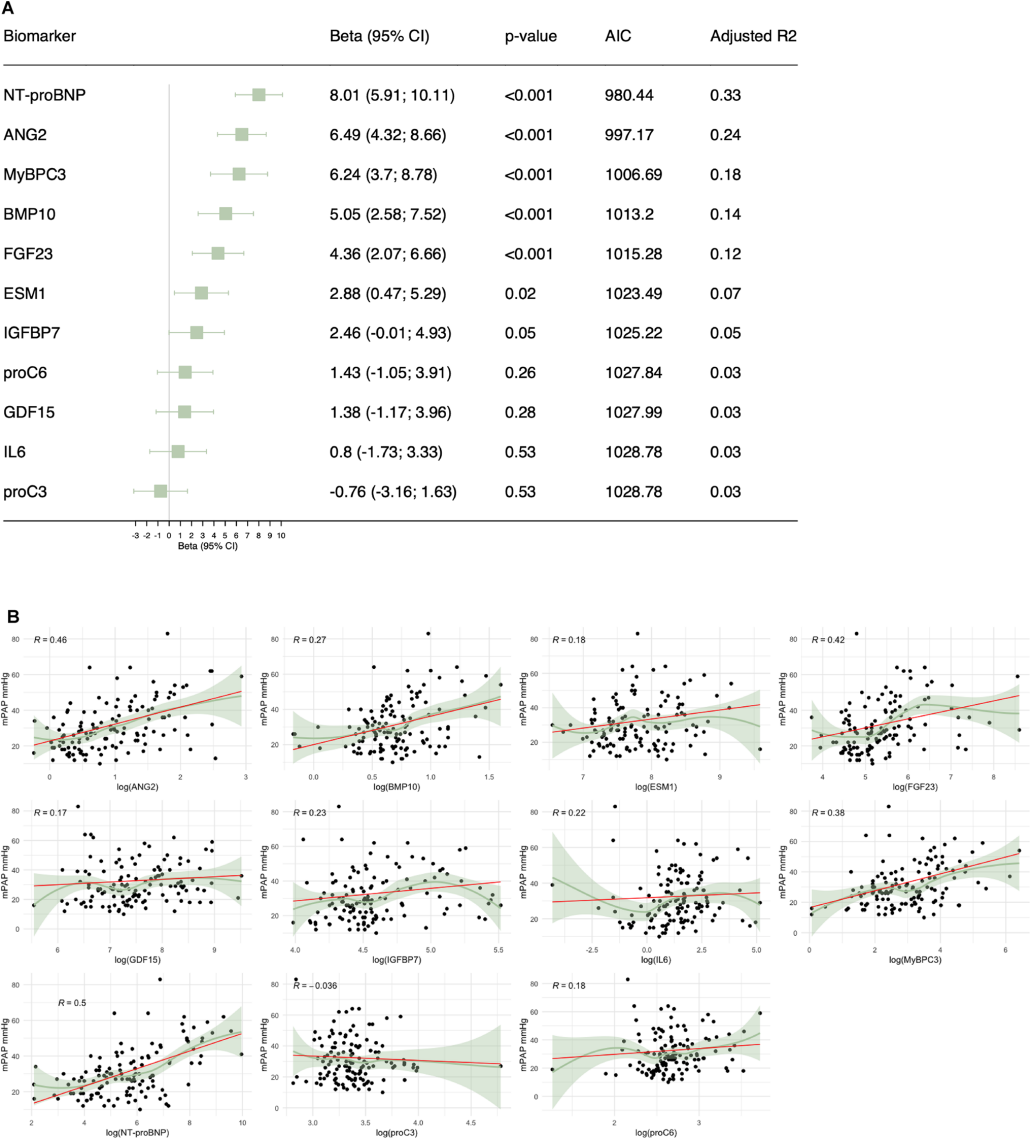
Association of biomarkers and mean pulmonary arterial pressure (mPAP). **A** Linear regression of the respective biomarker model adjusted for age, sex, and body mass index. All biomarkers are log-transformed and standardized. Models are sorted by β coefficients. *AIC* = Akaike information criterion. **B** Scatterplots of biomarkers and mPAP. R indicates the Spearman correlation coefficient. LOESS (locally estimated scatterplot smoothing) method is used to fit a smooth curve through the points of the scatterplot. Red line indicates linear-regression line of the age-, sex-, and body mass index-adjusted model for an “average” patient (mean age [66 years], most frequent sex category [female], mean body mass index [28 kg/m^2^])

We determined the association of candidate biomarkers with the hemodynamic parameter mPAP in linear-regression models. Results of adjusted models for age, sex, and BMI showed strong positive associations of NT-proBNP, ANG2, MyBPC3, BMP10, FGF23, and ESM1 with mPAP, in descending order **(**Fig. 2A**/B).** The highest adjusted R^2^ (0.33) was detected in the NT-proBNP model. This indicates that 33% of the variance in mPAP can be explained by this model. Results of the unadjusted and age- and sex-adjusted models are presented in Table S3.

**Fig. 3:**
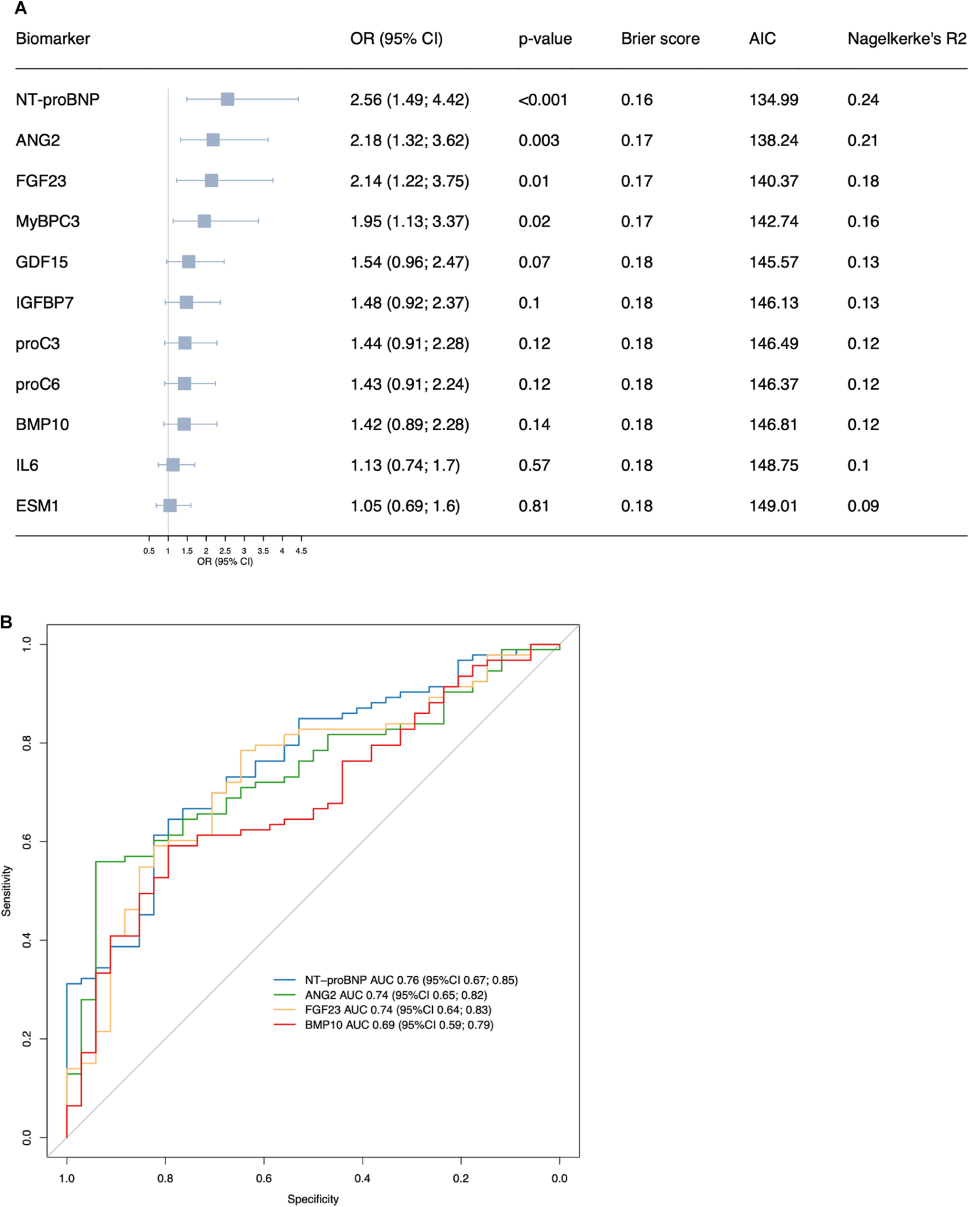
Association of biomarkers and pulmonary hypertension (PH). **A** Logistic regression of the respective biomarker model adjusted for age, sex, and body mass index for PH (yes/no). All biomarkers are log-transformed and standardized. Models are sorted by odds ratios (OR). *AIC* = Akaike information criterion. **B** Receiver operating characteristic (ROC) curves for PH (yes/no). Biomarker models adjusted for age, sex, and body mass index. Biomarker models are sorted by area under the curve (AUC)

If utilized for the association with PH, defined as mPAP > 20 mmHg, the models of NT-proBNP, ANG2, FGF23, and MyBPC3 adjusted for age, sex, and BMI showed strongly elevated ORs in descending order (Fig. 3A). Predicted probabilities of PH, calculated based on the age-, sex-, and BMI-adjusted logistic regression models for an “average” patient (mean age [66 years], most frequent sex category [female], mean BMI [28 kg/m^2^]) are shown in Fig. S5. Biomarker concentrations overall and stratified by PH are shown in Table S4. An additional analysis comparing hemodynamic parameters and biomarker levels between patients with PAH (*n* = 26) who were on PAH-specific therapy and those who were not (Table S5) did not show any strong differences between these groups. However, due to the very small sample size of the treated group, the statistical power of this analysis is highly limited. Receiver operating characteristics (ROC) analysis identified NT-proBNP as the best predictor of PH with an AUC of 0.76, followed by ANG2 and FGF23 (both AUC 0.74) (Fig. 3B). There was no evidence of any interaction of biomarkers with age or sex (p values for all interaction terms > 0.05), except for IGFBP7, which revealed an interaction with sex (*p* = 0.04) (data not shown). For the prediction of PH, a model consisting of ANG2, ESM1, GDF15, and NT-proBNP proved to be the best multibiomarker model **(**Table S6**)**. A likelihood ratio test between this best multibiomarker model and the model containing only NT-proBNP (best univariable model) was significant (*p* = 0.01), with an increase in AUC of 0.06 (0.77 vs. 0.71), a lower AIC (133 vs. 138), and a lower Brier score (0.16 vs. 0.18).

## Discussion

In this prospective cross-sectional study, which includes patients with a broad range of pulmonary hemodynamic profiles, we demonstrated hereto unknown associations of selected blood-based biomarkers with hemodynamic parameters measured during RHC. Specifically, we identified NT-proBNP, BMP10, ANG2, MyBPC3, and FGF23 as the top 5 candidate biomarkers associated with mPAP and PVR. Importantly, of the top 5 candidates, only BMP10 showed a strong association with high PVR (> 2 WU) and was not associated with PAWP. Thus, BMP10 indicates a higher mPAP based on higher PVR independent of PAWP. This distinctive behavior puts BMP10 in the unique position to discriminate the pre-capillary component from the post-capillary component of the pulmonary arterial pressure.

PH can result from different conditions, such as cardiac, pulmonary, thromboembolic, or miscellaneous diseases [1]. The underlying pathophysiology is complex and remains incompletely understood. There are three major pathogenetic factors in pulmonary arterial hypertension (PAH) which lead to an elevated pulmonary pressure and which are also present in most other forms of PH: vasoconstriction, thrombosis, and remodeling [5]. Remodeling is the major factor and involves all vascular cell types (endothelial cells, smooth muscle cells, and adventitial fibroblasts) [5]. The disturbed interaction is mediated by a large variety of growth factors, mitogens, cytokines, ion channels, receptors, neurotransmitters, and transcription factors [5].

BMP10 is a member of the transforming growth factor (TGF-β) superfamily. Its expression is mainly restricted to the right atrium, where it is expressed in right atrial cardiomyocytes [6–8]. Previous studies have shown that it is also excreted by the heart, making it measurable in the circulating blood, and thus also in venous blood samples [7]. This is a fundamental difference and key advantage of BMP10 as compared to other biomarkers of the right/left heart differentiation, such as the PITX2 transcription factor (paired‐like homeodomain transcription factor 2), which is not known to be released into the circulation [7, 9]. BMP10 exhibits unique specificity and affinity to the receptor complex composed of activin receptor-like kinase 1 (ALK1) and bone morphogenetic protein receptor type 2 (BMPR2), which is expressed in endothelial cells of the pulmonary vascular bed [10, 11]. By signaling through ALK1/BMPR2, BMP10 inhibits cell proliferation and thereby contributes to the tonic quiescence of the vascular endothelium [10–14]. Furthermore, BMP10 was found to directly act on vascular smooth muscle cells [15]. Together with bone morphogenetic protein 9 (BMP9), which is mostly expressed in the liver, there seems to be a BMP9/BMP10/ALK1/BMPR2 axis responsible for maintaining endothelial integrity and function [11]. A disruption of this axis by mutations, loss of circulating levels of ligands, or downregulation of receptors promotes endothelial dysfunction, increased vascular permeability, apoptosis, disordered angiogenesis, and consequently the development of PAH by vascular remodeling [11]. Mutations in the gene encoding BMPR2 are the main genetic cause of PAH [16, 17]. Mutations in ACVRL1, the gene for ALK1, are mainly causative of hereditary hemorrhagic telangiectasia and are sometimes associated with PAH [18, 19].

Our results demonstrate for the first time a strong association of serum BMP10 concentration with mPAP and PVR, independent from PAWP, in patients undergoing RHC for hemodynamic evaluation of suspected or established PH. These findings are thus in line with the involvement of the BMP10/ALK1/BMPR2 axis in pathologic vascular remodeling of pre-capillary arterioles. As the expression of BMP10 is mainly restricted to the right atrium and due to the high affinity of BMP10 to the receptor complex ALK1/BMPR2 in the pulmonary vasculature, BMP10 may be in the position to specifically reflect pathologic processes in the pre-capillary compartment of the lung circulation. Our results corroborate this assumption by finding no association of BMP10 with post-capillary hemodynamics (PAWP), yet a strong association with PVR. This is in vast contrast to NT-proBNP, ANG2, MyBPC3 and FGF23, which all showed strong associations with both pre- and post-capillary hemodynamics. In line with our findings, a previous study showed altered BMP10 concentrations in female PAH patients in comparison to female control subjects. However, no such altered BMP10 concentrations were found in male patients [20]. This could be explained by the fact that the aforementioned study measured ProBMP10 (pBMP10) concentrations, which is the prodomain-bound form of BMP10. It thus might not have the same characteristics. A commercial assay for BMP10 is currently not available, but it could be implemented in any hospital with routine blood analysis once a formal registration would be done.

NT-proBNP, a cardiac biomarker associated with cardiac wall stress and routinely used as a diagnostic tool in patients with heart failure, was associated with all hemodynamic parameters as well as the diagnosis of PH in our study. Notably, among our tested biomarkers, the best discriminative ability was shown by NT-proBNP with an AUC of 0.76, followed by ANG2 and FGF23 (both AUC 0.74). According to the guidelines, the measurement of NT-proBNP or BNP is recommended in patients with suspected PH as part of the diagnostic algorithm [1]. Our findings underline the importance of NT-proBNP measurement in the clinical workup of PH. However, NT-proBNP can be elevated in heart failure without PH and thus is not PH-specific [1]. The measurement of BMP10 in addition to NT-proBNP may therefore be useful to assess the pre-capillary component of PVR in a non-invasive setting. This might prove valuable for better stratification of PH patients as a higher PVR due to pre-capillary remodeling is associated with right heart failure and a worse prognosis [21]. Along this line, our group recently provided the first evidence of an additional value of BMP10 to NT-proBNP risk stratification in predicting all-cause death and major adverse cardiovascular events in patients with atrial fibrillation [22].

As recently demonstrated in previous studies, we found solid associations of ANG2 and FGF23 with pulmonary hemodynamics [23, 24]. ANG2 is synthesized by vascular endothelial cells and serves as autocrine-negative regulator of the quiescent resting endothelium [25]. In addition, ANG2 impairs venous thrombus resolution and thus might play a role in the pathogenesis of chronic thromboembolic pulmonary hypertension (CTEPH) [26]. In line with our findings, ANG2 was previously found to correlate with mPAP and PVR in patients with idiopathic PAH and patients with CTEPH [23, 27]. FGF23 is involved in phosphate metabolism and is a marker of renal dysfunction [28]. Higher levels of FGF23 were found in PH patients compared to healthy controls [24]. In addition, FGF23 patients in the highest FGF23 tertile had higher mPAP and PVR, which is compatible with our findings [24]. Similar to NT-proBNP, serum levels of ANG2 and FGF23 are also elevated in left heart failure [29, 30], which is in line with their strong association with PAWP in our study.

### Strengths and limitations

The key strength of our study was the standardized and simultaneous measurement of a large number of biomarkers during RHC. Moreover, PH diagnosis was made by two independent physicians.

The main limitations of our study were the relatively small sample size and the lack of a validation cohort. For the calculation of cardiac output according to Fick’s principle to determine PVR, oxygen uptake was derived from standard reference tables rather than being individually measured (indirect Fick method). Our results apply only to patients in sinus rhythm as we excluded patients who had experienced atrial fibrillation in the previous 6 months. Lastly, most study participants were of European origin, which limits the generalizability of our findings.

## Conclusion

Several biomarkers were strongly associated with hemodynamic parameters measured during RHC in our study. In contrast to other biomarkers, BMP10 is most strongly associated with PVR and shows no association with PAWP. It thus reflects the pre-capillary component of PH. The measurement of BMP10 might prove useful in the diagnosis and risk stratification of patients with suspected or established PH in addition to NT-proBNP. Additional research is needed before biomarkers can be routinely used to guide or even replace RHC in the clinical setting. Future studies should aim to evaluate the use of these biomarkers in combination with non-invasive imaging techniques.

## Supplementary Information

Below is the link to the electronic supplementary material.

Supplementary file1 (DOCX 18206 KB)

## Data Availability

The data that support the findings of this study are not openly available due to reasons of sensitivity and are available from the corresponding author upon reasonable request.
