# Supplementary material for: BMP10 reflects pre-capillary pulmonary hemodynamics: association of biomarkers and hemodynamic parameters in pulmonary hypertension
Source: Clin Res Cardiol. 2024 Sep 19;114(2):239–50. doi: 10.1007/s00392-024-02546-8 (PMC11839876; doi:10.1007/s00392-024-02546-8)
Supplement: Supplementary file 1 — Supplementary file1 (DOCX 18206 KB) [file 392_2024_2546_MOESM1_ESM.docx]

SUPPLEMENT

BMP10 reflects Pre-capillary Pulmonary Hemodynamics:

Association of Biomarkers and Hemodynamic Parameters in Pulmonary Hypertension

| Content |  | Page |
| --- | --- | --- |
| Figure S1 | Pulmonary hypertension diagnosis and type | 2 |
| Figure S2 | Pulmonary hypertension clinical classification | 3 |
| Figure S3 | Scatterplots of biomarkers and pulmonary vascular resistance (PVR) | 4 |
| Figure S4 | Scatterplots of biomarkers and pulmonary arterial wedge pressure (PAWP) | 5 |
| Figure S5 | Predicted probabilities of pulmonary hypertension | 6 |
| Table S1 | Association of biomarkers and pulmonary vascular resistance (PVR) | 7 |
| Table S2 | Association of biomarkers and pulmonary arterial wedge pressure (PAWP) | 9 |
| Table S3 | Association of biomarkers and mean pulmonary arterial pressure (mPAP) | 11 |
| Table S4 | Biomarker concentration overall and stratified by pulmonary hypertension | 13 |
| Table S5 | Hemodynamic parameters and biomarker concentration in patients with pulmonary arterial hypertension (PAH) stratified by PAH-medication | 14 |
| Table S6 | Variable selection for pulmonary hypertension (yes/no) | 15 |

Figure S1: Pulmonary hypertension diagnosis and type

Pulmonary hypertension diagnosis and type were defined according to the European Society of Cardiology (ESC) guidelines of 2022.

Figure S2: Pulmonary hypertension clinical classification

Pulmonary hypertension (PH) clinical classification according to the European Society of Cardiology (ESC) guidelines of 2022: group 1 = pulmonary arterial hypertension, group 2 = PH associated with left heart disease, group 3 = PH associated with lung diseases and/or hypoxia, group 4 = PH associated with pulmonary artery obstruction, group 5 = PH with unclear and/or multifactorial mechanisms.

Figure S3: Scatterplots of biomarkers and pulmonary vascular resistance (PVR)

R indicates the Spearman correlation coefficient. LOESS (locally estimated scatterplot smoothing) method is used to fit a smooth curve through the points of the scatterplot. Red line indicates linear regression line of the age-, sex-, and body mass index-adjusted model for an “average” patient (mean age [66 years], most frequent sex category [female], mean body mass index [28 kg/m2]).


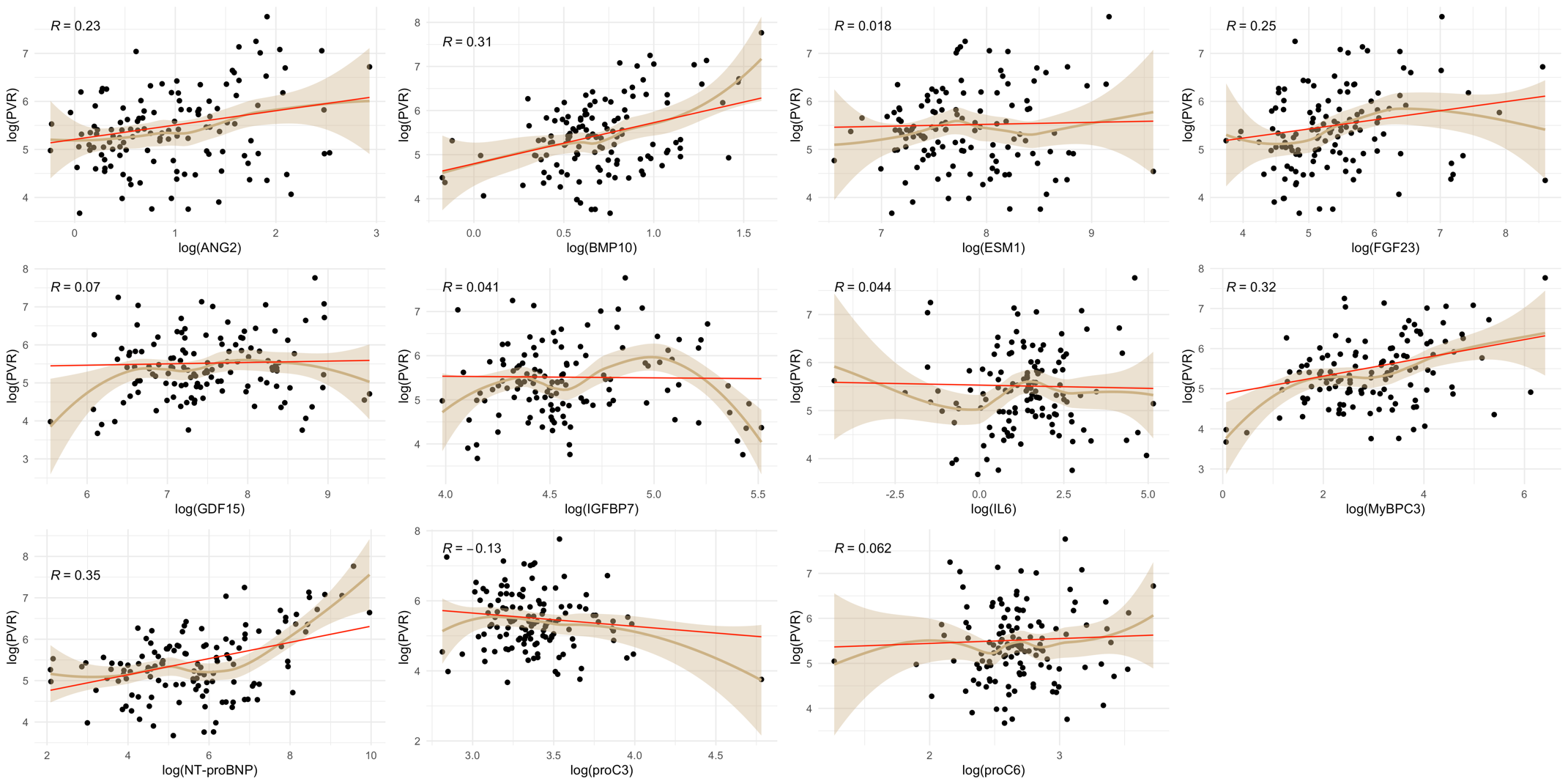


Figure S4: Scatterplots of biomarkers and pulmonary arterial wedge pressure (PAWP)

R indicates the Spearman correlation coefficient. LOESS (locally estimated scatterplot smoothing) method is used to fit a smooth curve through the points of the scatterplot. Red line indicates linear regression line of the age-, sex-, and body mass index-adjusted model for an “average” patient (mean age [66 years], most frequent sex category [female], mean body mass index [28 kg/m2]).


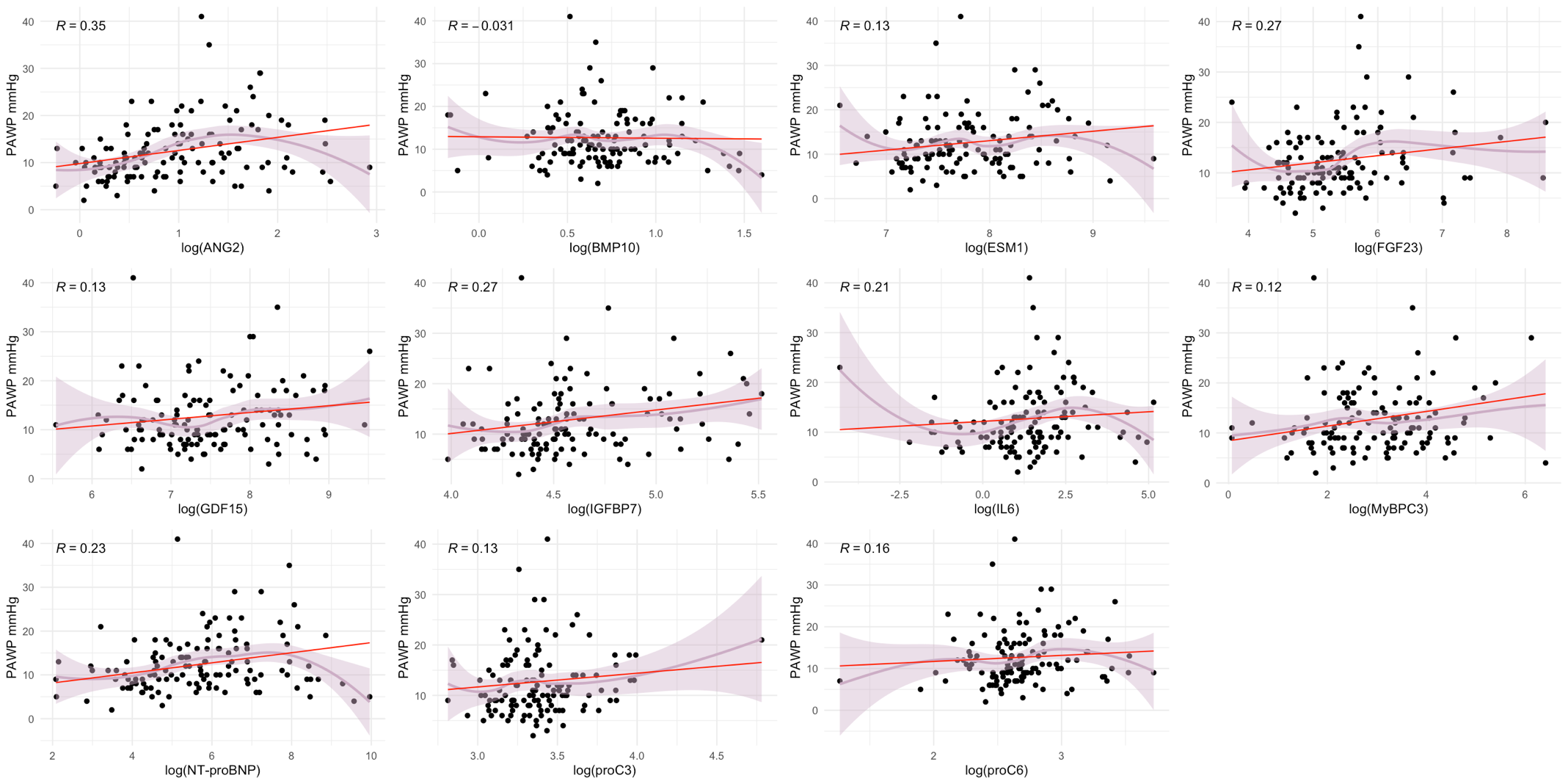


Figure S5: Predicted probabilities of pulmonary hypertension

Probabilities for pulmonary hypertension (PH) are calculated based on the age-, sex-, and body mass index-adjusted logistic regression models of the respective biomarker for an “average” patient (mean age [66 years], most frequent sex category [female], mean body mass index [28 kg/m2]).

**
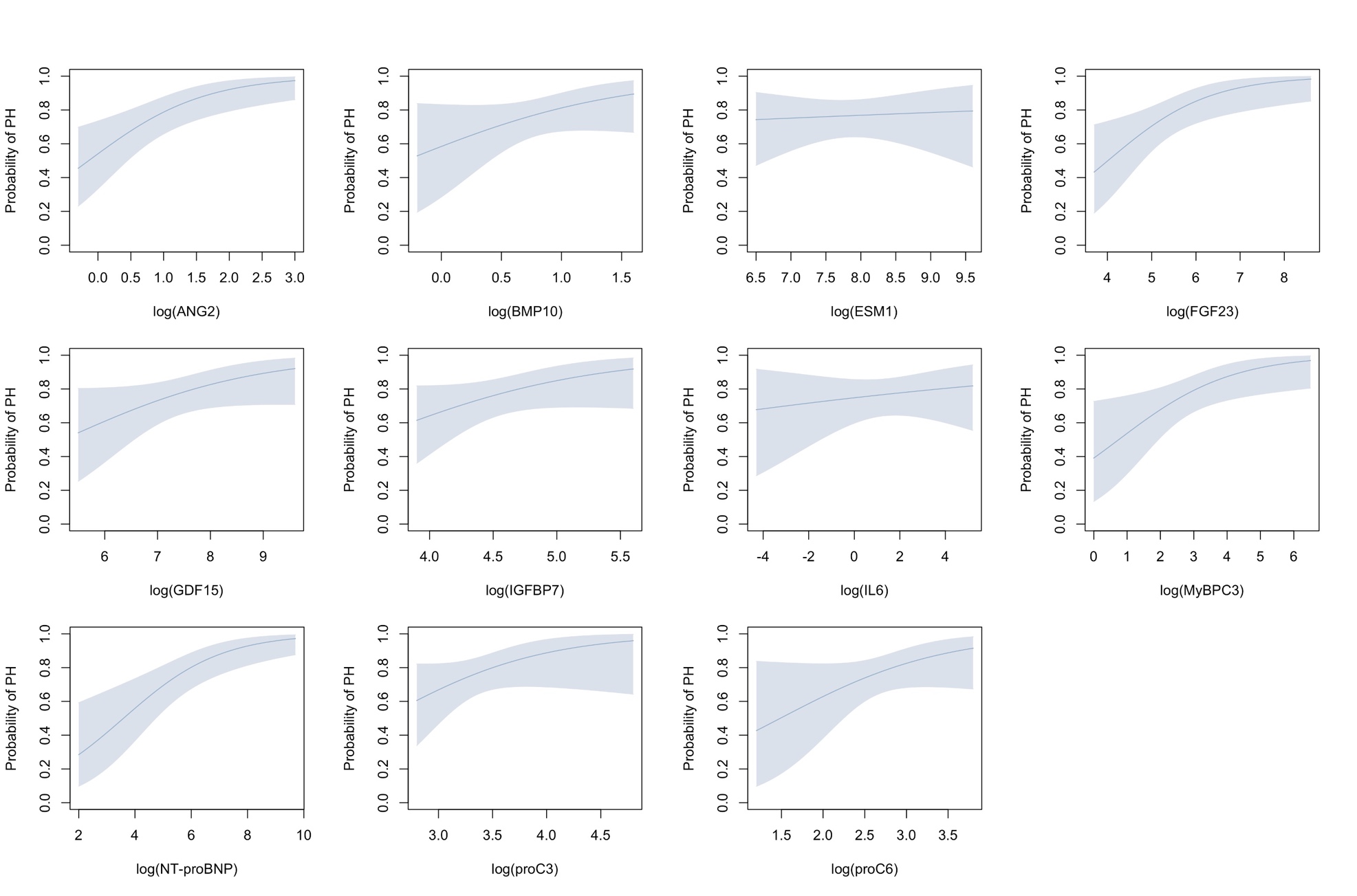
**

| Table S1: Association of biomarkers and pulmonary vascular resistance (PVR) | | | | | |
| --- | --- | --- | --- | --- | --- |
| **Linear regression** | **Univariable unadjusted model** |  | **Age- and sex-adjusted model** |  | **Age-, sex- and BMI-adjusted model** |
|  | Standardized β (95% CI), p-value  *Multiple R^2^*  AIC |  | Standardized β (95% CI), p-value  *Adjusted R^2^*  AIC |  | Standardized β (95% CI), p-value  *Adjusted R^2^*  AIC |
| ANG2 | 0.23 (0.09; 0.37), 0.001  *0.08*  300.65 |  | 0.20 (0.07; 0.34), 0.003  *0.17*  288.93 |  | 0.20 (0.07; 0.34), 0.003  *0.16*  290.92 |
| BMP10 | 0.34 (0.21; 0.47), <0.001  *0.18*  286.45 |  | 0.29 (0.15; 0.42), <0.001  *0.21*  281.65 |  | 0.29 (0.15; 0.43), <0.001  *0.21*  283.05 |
| ESM1 | 0.06 (-0.08 0.21), 0.38  *0.01*  310.21 |  | 0.02 (-0.12; 0.16), 0.78  *0.11*  297.85 |  | 0.02 (-0.12; 0.16), 0.75  *0.10*  299.77 |
| FGF23 | 0.18 (0.04; 0.32), 0.01  *0.05*  304.24 |  | 0.16 (0.03; 0.30), 0.02  *0.15*  291.91 |  | 0.16 (0.03; 0.30), 0.02  *0.14*  293.89 |
| GDF15 | 0.09 (-0.05; 0.23), 0.21  *0.01*  309.37 |  | 0.03 (-0.12; 0.17), 0.69  *0.11*  297.77 |  | 0.03 (-0.12; 0.17), 0.71  *0.10*  299.73 |
| IGFBP7 | 0.02 (-0.12; 0.17), 0.77  *0.001*  310.92 |  | -0.01 (-0.15; 0.13), 0.88  *0.10*  297.90 |  | -0.01 (-0.15; 0.13), 0.86  *0.10*  299.85 |
| IL6 | 0.03 (-0.12; 0.17), 0.69  *0.001*  310.84 |  | -0.02 (-0.16; 0.12), 0.81  *0.10*  297.87 |  | -0.02 (-0.16; 0.12), 0.78  *0.10*  299.80 |
| MyBPC3 | 0.31 (0.18; 0.44), <0.001  *0.15*  290.30 |  | 0.26 (0.11; 0.41), 0.001  *0.18*  286.62 |  | 0.26 (0.11; 0.41), 0.001  *0.17*  288.51 |
| NT-proBNP | 0.37 (0.25; 0.50), <0.001  *0.21*  280.74 |  | 0.31 (0.18; 0.45), <0.001  *0.24*  276.71 |  | 0.32 (0.19; 0.45), <0.001  *0.24*  278.31 |
| proC3 | -0.14 (-0.28; 0.004), 0.06  *0.03*  307.28 |  | -0.10 (-0.23; 0.03), 0.14  *0.12*  295.64 |  | -0.10 (-0.24; 0.03), 0.14  *0.11*  297.55 |
| proC6 | 0.07 (-0.08; 0.21), 0.35  *0.01*  310.12 |  | 0.04 (-0.10; 0.18), 0.58  *0.11*  297.61 |  | 0.04 (-0.10; 0.18), 0.60  *0.10*  299.59 |

n = 127. All biomarkers are log-transformed and standardized. The outcome pulmonary vascular resistance (PVR) was log-transformed. AIC = Akaike information criterion, BMI = body mass index.

| Table S2: Association of biomarkers and pulmonary arterial wedge pressure (PAWP) | | | | | |
| --- | --- | --- | --- | --- | --- |
| **Linear regression** | **Univariable unadjusted model** |  | **Age- and sex-adjusted model** |  | **Age-, sex-, and BMI-adjusted model** |
|  | Standardized β (95% CI), p-value  *Multiple R^2^*  AIC |  | Standardized β (95% CI), p-value  *Adjusted R^2^*  AIC |  | Standardized β (95% CI), p-value  *Adjusted R^2^*  AIC |
| ANG2 | 1.99 (0.90; 3.08), <0.001  *0.09*  822.59 |  | 2.08 (0.99; 3.18), <0.001  *0.09*  823.40 |  | 1.89 (0.84; 2.95), <0.001  *0.16*  814.25 |
| BMP10 | -0.53 (-1.67; 0.60), 0.35  *0.01*  834.26 |  | -0.37 (-1.60; 0.86), 0.55  *-0.01*  836.92 |  | -0.10 (-1.29; 1.08), 0.86  *0.08*  826.70 |
| ESM1 | 0.62 (-0.50; 1.74), 0.27  *0.01*  833.91 |  | 0.76 (-0.37; 1.89), 0.19  *0.005*  835.47 |  | 1.16 (0.07; 2.25), 0.04  *0.11*  822.16 |
| FGF23 | 1.42 (0.32; 2.52), 0.01  *0.05*  828.71 |  | 1.48 (0.38; 2.58), 0.01  *0.05*  830.20 |  | 1.22 (0.15; 2.30), 0.03  *0.12*  821.52 |
| GDF15 | 0.81 (-0.30; 1.92), 0.15  *0.02*  833.04 |  | 1.20 (0.01; 2.39), 0.048  *0.02*  833.21 |  | 1.06 (-0.08; 2.20), 0.07  *0.10*  823.22 |
| IGFBP7 | 1.49 (0.38; 2.59), 0.01  *0.05*  828.11 |  | 1.74 (0.60; 2.88), 0.003  *0.06*  828.15 |  | 1.58 (0.49; 2.68), 0.01  *0.14*  818.49 |
| IL6 | 0.64 (-0.50; 1.78), 0.27  *0.01*  833.90 |  | 0.84 (-0.33; 2.01), 0.16  *0.01*  835.22 |  | 0.57 (-0.57; 1.70), 0.32  *0.09*  825.72 |
| MyBPC3 | 0.79 (-0.32; 1.90), 0.16  *0.02*  833.15 |  | 1.58 (0.31; 2.86), 0.02  *0.04*  831.22 |  | 1.65 (0.44; 2.87), 0.01  *0.13*  819.43 |
| NT-proBNP | 1.29 (0.18; 2.40), 0.02  *0.04*  829.83 |  | 1.70 (0.54; 2.85), 0.004  *0.06*  828.86 |  | 1.87 (0.77; 2.97), <0.001  *0.16*  815.37 |
| proC3 | 0.90 (-0.21; 2.00), 0.11  *0.02*  832.53 |  | 0.83 (-0.29; 1.95), 0.14  *0.01*  835.06 |  | 0.73 (-0.34; 1.81), 0.18  *0.09*  824.84 |
| proC6 | 0.74 (-0.39; 1.87), 0.20  *0.01*  833.43 |  | 0.87 (-0.27; 2.02), 0.13  *0.01*  834.94 |  | 0.51 (-0.62; 1.63), 0.37  *0.08*  825.91 |

n = 127. All biomarkers are log-transformed and standardized. AIC = Akaike information criterion, BMI = body mass index.

| Table S3: Association of biomarkers and mean pulmonary arterial pressure (mPAP) | | | | | |
| --- | --- | --- | --- | --- | --- |
| **Linear regression** | **Univariable unadjusted model** |  | **Age- and sex-adjusted model** |  | **Age-, sex- and BMI-adjusted model** |
|  | Standardized β (95% CI), p-value  *Multiple R^2^*  AIC |  | Standardized β (95% CI), p-value  *Adjusted R^2^*  AIC |  | Standardized β (95% CI), p-value  *Adjusted R^2^*  AIC |
| ANG2 | 6.82 (4.67; 8.98), <0.001  *0.24*  995.51 |  | 6.69 (4.51; 8.86), <0.001  *0.23*  998.19 |  | 6.49 (4.32; 8.66), <0.001  *0.24*  997.17 |
| BMP10 | 4.72 (2.40; 7.03), <0.001  *0.12*  1014.61 |  | 4.63 (2.11; 7.14), <0.001  *0.09*  1018.55 |  | 5.05 (2.58; 7.52), <0.001  *0.14*  1013.20 |
| ESM1 | 2.52 (0.14; 4.91), 0.04  *0.03*  1025.79 |  | 2.31 (-0.11; 4.73), 0.06  *0.02*  1027.93 |  | 2.88 (0.47; 5.29), 0.02  *0.07*  1023.49 |
| FGF23 | 4.76 (2.47; 7.04), <0.001  *0.12*  1013.96 |  | 4.64 (2.35; 6.93), <0.001  *0.11*  1016.00 |  | 4.36 (2.07; 6.66), <0.001  *0.12*  1015.28 |
| GDF15 | 1.56 (-0.84; 3.95), 0.20  *0.01*  1028.50 |  | 1.56 (-1.01; 4.14), 0.23  *0.008*  1030.10 |  | 1.38 (-1.17; 3.96), 0.28  *0.03*  1027.99 |
| IGFBP7 | 2.50 (0.09; 4.90), 0.04  *0.03*  1025.98 |  | 2.66 (0.17; 5.15), 0.04  *0.03*  1027.04 |  | 2.46 (-0.01; 4.93), 0.05  *0.05*  1025.22 |
| IL6 | 1.24 (-1.22; 3.69), 0.32  *0.008*  1029.17 |  | 1.15 (-1.38; 3.68), 0.37  *0.003*  1030.74 |  | 0.80 (-1.73; 3.33), 0.53  *0.03*  1028.78 |
| MyBPC3 | 5.10 (2.86; 7.34), <0.001  *0.14*  1011.03 |  | 6.15 (3.56; 8.74), <0.001  *0.15*  1010.57 |  | 6.24 (3.70; 8.78), <0.001  *0.18*  1006.69 |
| NT-proBNP | 7.56 (5.52; 9.59), <0.001  *0.30*  984.58 |  | 7.75 (5.59; 9.91), <0.001  *0.29*  988.06 |  | 8.01 (5.91; 10.11), <0.001  *0.33*  980.44 |
| proC3 | -0.90 (-3.30; 1.50), 0.46  *0.004*  1029.61 |  | -0.63 (-3.05; 1.80), 0.61  *-0.002*  1031.31 |  | -0.76 (-3.16; 1.63), 0.53  *0.03*  1028.78 |
| proC6 | 1.90 (-0.52; 4.32), 0.12  *0.02*  1027.74 |  | 1.88 (-0.58; 4.33), 0.13  *0.01*  1029.24 |  | 1.43 (-1.05; 3.91), 0.26  *0.03*  1027.84 |

n = 127. All biomarkers are log-transformed and standardized. AIC = Akaike information criterion, BMI = body mass index.

| **Table S4: Biomarker concentration overall and stratified by pulmonary hypertension** | | | | |
| --- | --- | --- | --- | --- |
| **Biomarker** | **Overall** | **Pulmonary hypertension** | | |
|  |  | **No** | **Yes** | **p-value** |
| *Number of patients* | *127 (100)* | *34 (27)* | *93 (73)* |  |
| ANG2, ng/mL | 2.38 [1.57, 4.20] | 1.61 [1.34, 2.30] | 2.80 [1.78, 4.80] | <0.001 |
| BMP10, ng/mL | 1.96 [1.68, 2.33] | 1.88 [1.62, 2.18] | 1.96 [1.69, 2.42] | 0.18 |
| ESM1, pg/mL | 2250.54 [1637.19, 3570.30] | 2259.21 [1576.63, 3280.98] | 2250.54 [1657.00, 3650.74] | 0.77 |
| FGF23, pg/mL | 199.35 [121.60, 314.17] | 127.10 [103.41, 170.20] | 235.76 [156.53, 362.15] | <0.001 |
| GDF15, pg/mL | 1593.80 [1083.99, 3058.30] | 1419.72 [758.54, 1994.72] | 1791.52 [1170.10, 3451.97] | 0.03 |
| IGFBP7, ng/mL | 91.58 [79.63, 117.04] | 90.17 [71.79, 98.79] | 93.28 [81.80, 124.29] | 0.06 |
| IL6, pg/mL | 4.13 [1.85, 9.26] | 2.38 [1.21, 5.26] | 4.57 [2.41, 10.50] | 0.03 |
| MyBPC3, pg/mL | 19.10 [9.17, 41.58] | 12.92 [5.64, 25.43] | 23.63 [9.98, 46.08] | 0.02 |
| NT-proBNP, pg/mL | 291.37 [95.21, 740.53] | 97.27 [54.11, 304.73] | 356.89 [127.93, 981.45] | <0.001 |
| proC3, ng/mL | 28.86 [24.78, 33.47] | 28.36 [24.92, 32.14] | 28.86 [24.73, 34.16] | 0.34 |
| proC6, ng/mL | 14.39 [12.17, 17.20] | 12.85 [11.43, 14.83] | 14.48 [12.81, 17.55] | 0.02 |

Values are given as median [interquartile range]. ANG2 = Angiopoietin 2, BMP10 = Bone morphogenetic protein 10, ESM1 = Endothelial cell specific molecule 1 / endocan, FGF23 = Fibroblast growth factor 23, GDF15 = Growth differentiation factor 15, IGFBP7 = Insulin-like growth factor-binding protein 7, IL6 = Interleukin 6, MyBPC3 = Myosin binding protein C3, NT-proBNP = N-terminal prohormone of B-type natriuretic peptide, proC3 = N-terminal type III collagen propeptide, proC6 = fragment of C-terminal type VIa3 collagen / endotrophin.

| **Table S5: Hemodynamic parameters and biomarker concentration in patients with pulmonary arterial hypertension (PAH) stratified by PAH-medication (incl. endothelin receptor antagonists, PDE5 inhibitors, guanylate cyclase stimulators, and prostacyclin analogs)** | | | |
| --- | --- | --- | --- |
|  | **Medication for PAH** | |  |
|  | **Yes** | **No** | **p-value** |
| *Number of patients* | *20* | *6* |  |
| Right heart catheterization: |  |  |  |
| - Mean pulmonary arterial pressure (mPAP), mmHg (mean (SD)) | 42.15 (10.90) | 48.33 (21.19) | 0.34 |
| - Pulmonary vascular resistance (PVR), dyn*S*cm^-5^ (median [IQR]) | 433.11 [314.21, 675.12] | 445.50 [230.86, 975.33] | 0.95 |
| - Pulmonary arterial wedge pressure (PAWP), mmHg (mean (SD)) | 12.35 (6.58) | 14.00 (3.03) | 0.56 |
| Biomarker: |  |  |  |
| - ANG2, ng/mL (median [IQR]) | 3.59 [1.53, 5.85] | 4.45 [2.09, 7.46] | 0.50 |
| - BMP10, ng/mL (median [IQR]) | 1.93 [1.69, 2.37] | 2.91 [2.40, 3.34] | 0.07 |
| - ESM1, pg/mL (median [IQR]) | 2124.97 [1699.95, 3475.19] | 3472.02 [2645.94, 4195.81] | 0.10 |
| - FGF23, pg/mL (median [IQR]) | 237.54 [131.69, 316.15] | 396.44 [140.07, 598.44] | 0.50 |
| - GDF15, pg/mL (median [IQR]) | 3023.20 [1317.52, 4395.05] | 1195.57 [802.30, 2467.51] | 0.18 |
| - IGFBP7, ng/mL (median [IQR]) | 116.23 [79.46, 144.42] | 88.46 [77.46, 118.92] | 0.54 |
| - IL6, pg/mL (median [IQR]) | 5.86 [3.91, 11.85] | 8.85 [1.33, 14.57] | 0.93 |
| - MyBPC3, pg/mL (median [IQR]) | 41.58 [10.42, 70.41] | 16.23 [11.75, 26.82] | 0.50 |
| - NT-proBNP, pg/mL (median [IQR]) | 359.84 [95.46, 3309.74] | 789.72 [420.82, 973.36] | 0.72 |
| - proC3, ng/mL (median [IQR]) | 28.56 [23.84, 34.42] | 28.00 [24.34, 35.18] | 1.00 |
| - proC6, ng/mL (median [IQR]) | 15.83 [12.48, 19.52] | 13.48 [10.27, 14.84] | 0.20 |

ANG2 = Angiopoietin 2, BMP10 = Bone morphogenetic protein 10, ESM1 = Endothelial cell specific molecule 1 / endocan, FGF23 = Fibroblast growth factor 23, GDF15 = Growth differentiation factor 15, IGFBP7 = Insulin-like growth factor-binding protein 7, IL6 = Interleukin 6, MyBPC3 = Myosin binding protein C3, NT-proBNP = N-terminal prohormone of B-type natriuretic peptide, proC3 = N-terminal type III collagen propeptide, proC6 = fragment of C-terminal type VIa3 collagen / endotrophin.

| Table S6: Variable selection for pulmonary hypertension (yes/no) | | |
| --- | --- | --- |
| **Logistic regression** | **Selected multibiomarker model** | *VIF* |
|  | OR (95% CI) |  |
| ANG2 | 1.73 (0.92; 3.27) | *1.45* |
| BMP10 | - | *-* |
| ESM1 | 0.49 (0.28; 0.86) | *1.51* |
| FGF23 | - | *-* |
| GDF15 | 1.61 (0.95; 2.74) | *1.20* |
| IGFBP7 | - | *-* |
| IL6 | - | *-* |
| MyBPC3 | - | *-* |
| NT-proBNP | 2.19 (1.16; 4.14) | *1.45* |
| proC3 | - | *-* |
| proC6 | - | *-* |
| **AIC** | 133.20 |  |
| **AUC (95% CI)** | 0.77 (0.67; 0.85) |  |
| **Brier score** | 0.16 |  |
| **Nagelkerke's R^2^** | 0.25 |  |

n = 127. All biomarkers are log-transformed and standardized. AIC = Akaike information criterion, AUC = area under the curve, OR = odds ratio, VIF = variance inflation factor.
